# High phosphate intake induces bone loss in nephrectomized thalassemic mice

**DOI:** 10.1371/journal.pone.0268732

**Published:** 2022-05-27

**Authors:** Sasithorn Wanna-udom, Chainarong Luesiripong, Nithidol Sakunrangsit, Piyanuch Metheepakornchai, Sitthichai Intharamonthian, Saovaros Svasti, Matthew B. Greenblatt, Asada Leelahavanichkul, Sutada Lotinun

**Affiliations:** 1 Faculty of Dentistry, Department of Physiology, Center of Excellence in Skeletal Disorders and Enzyme Reaction Mechanism, Chulalongkorn University, Bangkok, Thailand; 2 Faculty of Science, Thalassemia Research Center, Institute of Molecular Biosciences, Department of Biochemistry, Mahidol University, Bangkok, Thailand; 3 Department of Pathology and Laboratory Medicine, Weill Cornell Medicine, Research Division, Hospital for Special Surgery, New York, NY, United States of America; 4 Faculty of Medicine, Division of Immunology, Department of Microbiology, Chulalongkorn University, Bangkok, Thailand; University of Vermont, UNITED STATES

## Abstract

Although patients with either β-thalassemia or chronic kidney disease (CKD) clinically correlate with severe osteoporosis, the mechanism by which CKD exposed to high phosphate affects bone turnover has not been characterized in β-thalassemia. We aimed to determine the effects of renal insufficiency on high phosphate intake induced changes in bone metabolism after 5/6^th^ nephrectomy in hemizygous *β-globin* knockout (BKO) mice. Male BKO mice manifested severe anemia and osteopenia. Nephrectomy induced renal fibrosis and reduced renal function as assessed by increased serum urea nitrogen levels. Moreover, nephrectomy increased bone turnover leading to bone loss in wild type (WT) but not BKO mice. In nephrectomized BKO, PBS in drinking water induced hyperphosphatemia, and hypercalcemia along with osteopenia in both cancellous and cortical bone. Histomorphometric analysis confirmed reduced cancellous bone volume due to decreased bone formation rate, osteoblast number and osteoclast number. The mRNA levels for *Alpl*, *Sp7*, *Kl*, *Tnfsf11*, and *Tnfsf11/Tnfrsf11b* were decreased in nephrectomized BKO mice drinking PBS. Interestingly, *Fgf23*, a bone-derived hormone produced by osteocytes and osteoblasts in response to hyperphosphatemia, were remarkably increased in nephrectomized BKO mice following PBS intake. Serum FGF23 and erythropoietin levels were markedly elevated in BKO mice. Nephrectomy decreased serum erythropoietin but not FGF23 levels. Hyperphosphatemia in BKO mice increased serum erythropoietin, FGF23, and PTH levels, nominating these factors as candidate mediators of bone loss in thalassemic mice with CKD during phosphate retention.

## Introduction

Hyperphosphatemia, abnormally high serum phosphate levels, results from increased dietary phosphate intake, an inability of kidney to excrete phosphate, and a shift of intracellular phosphate to extracellular fluid. In chronic kidney disease (CKD), phosphate retention begins at early stage of disease when the glomerular infiltration rate (GFR) decreases and less phosphate is excreted. When the GFR falls toward end-stage renal disease, hyperphosphatemia develops and is associated with secondary hyperparathyroidism and metabolic bone disease. CKD-mineral bone disorder (CKD-MBD) is characterized by abnormalities of bone and mineral metabolism and bone fragility together with increased FGF23, and secondary hyperparathyroidism in CKD patients on dialysis [1]. Hyperphosphatemia contributes to the development of CKD-MBD and early treatment of phosphate retention may prevent the skeletal manifestation of disease.

Three hormones, 1,25(OH)_2_D_3_, PTH, and FGF23 negatively regulate phosphate homeostasis and delays the occurrence of hyperphosphatemia [2]. In CKD patients, FGF23, a phosphaturic hormone secreted primarily by osteocytes, is increased, regulating renal phosphate and vitamin D metabolism. FGF23 in conjunction with Klotho, its co-receptor, induces phosphate excretion by reducing luminal sodium-dependent phosphate transporters in proximal renal tubule to maintain mineral homeostasis. It also inhibits renal 1α-hydroxylase activity to stimulate 1,25(OH)_2_D_3_ production, leading to a disturbance in bone homeostasis. This decrease in serum 1,25(OH)_2_D_3_ results in decreased intestinal calcium absorption, hypocalcemia, and hyperparathyroidism which, in turn, enhances phosphate excretion. PTH is a uremic toxin [3] and elevated PTH levels in CKD patients induces a pro-inflammatory response, and increases bone turnover. As CKD progresses to end-stage renal disease, phosphate handling becomes dysregulated. Compensatory mechanisms become insufficient to maintain phosphate homeostasis and prevent hyperphosphatemia in CKD stages 4 and 5 [4].

β-thalassemia, is characterized by partial or complete lack of β-globin chain synthesis due to point mutations of *β-globin* gene located on chromosome 11 in humans. Lack of normal β-globin production leads to excessive α-globin accumulation and precipitation, resulting in erythroid cell apoptosis and ineffective erythropoiesis [5]. Patients with β-thalassemia are anemic and have red blood cell morphologic changes such as microcytosis, hypochromia, anisocytosis, and poikilocytosis, hepatosplenomegaly and bone deformity [6,7]. β-thalassemia major, also known as Cooley anemia is the most severe form of the disease and is characterized by expansion of erythroid progenitors, ineffective erythropoiesis and extramedullary hematopoiesis. Previous studies revealed that proximal tubular damage was observed in patients with β-thalassemia major [8], and glomerular hyperfiltration was found in transfusion-independent thalassemia intermedia patients [9]. Additionally, iron overload from long-term blood transfusion-dependent thalassemia leads to much rapid worsening of CKD progression.

Previous studies have reported the association between thalassemia and osteoporosis due to bone marrow expansion from ineffective erythropoiesis, endocrine dysfunction and iron overload [6]. An imbalance between insufficient osteoblastic bone formation and enhanced osteoclastic bone resorption contributes to low bone mass in patients with thalassemia. Our previous study indicated that nephrectomy increased bone turnover, leading to cancellous and cortical bone loss in wild type (WT) [10]. Heterozygous *β-globin* knockout (BKO) mice, mouse model for β-thalassemia, were osteopenic; however, 5/6^th^ nephrectomy did not induced cancellous bone loss, possibly due to decreased serum erythropoietin levels. In addition, nephrectomy to induce renal failure did not lead to high serum phosphate in both WT and BKO mice.

To investigate the pathophysiology of CKD and the mechanism by which phosphate retention caused osteopenia in thalassemic mice, high phosphate intake model was performed. BKO mice and WT littermate were 5/6^th^ nephrectomized to induce CKD and fed phosphate buffered saline (PBS) for 4 months. We found that nephrectomy induced cancellous and cortical bone loss due to increased bone formation and bone resorption in WT but not BKO mice. High phosphate load reduced cancellous bone volume, cortical thickness and bone mineral density in nephrectomized BKO mice compared to nephrectomized BKO mice. The *Fgf23* mRNA levels were dramatically increased without any change in other bone formation and bone resorption markers. BKO mice markedly increased serum erythropoietin and FGF23. Serum FGF23 was highest in nephrectomized BKO fed PBS possibly due to increased serum PTH level.

## Materials and methods

### Animals

Male BKO mice and WT controls on C57BL/6 background were obtained from the Thalassemia Research Center, Institute of Molecular Biosciences, Mahidol University. The BKO mice exhibited a phenotype similar to that of human β-thalassemia intermedia. All methods were performed in accordance with the relevant guidelines and regulations and approved by the Institutional Animal Care and Use Committee at Faculty of Medicine, Chulalongkorn University (IACUC approval number 012/2562). Mice were housed at room temperature under a 12:12 h light-dark cycle. They were fed rodent chow (C.P. mice feed, Perfect Companion Group Co., Ltd., stock number 082, Thailand) and reverse osmosis water ad libitum.

The heterozygous BKO mice and WT littermates were generated and genotyping was performed by proxy through the morphologic examination of blood smears. Seven weeks old males were divided into 6 groups; sham WT, sham BKO, nephrectomized WT, nephrectomzied BKO, nephrectomized WT+PBS, and nephrectomized BKO+PBS. To generate the CKD mice, the 5/6^th^ nephrectomy model was performed using a two-step surgical procedure under isoflurane anesthesia to alleviate suffering. During the first step, the upper and lower poles or two-thirds of the left kidney were resected. Microfibrillar collagen hemostasis (Avitene, Davol, Cranston, RI) was used to control bleeding. After a week of the recovery period, a second surgery was performed to remove the entire right kidney. Mice were monitored until they were fully recovered and tramadol (25 mg/kg), an analgesic, was administered every 12 hours for 3 days. In the sham group, the incisions were performed without kidney removal. For PBS groups, mice were fed with 1X PBS (137 mM NaCl, 2.7 mM KCl, 10 mM Na_2_HPO_4_ and 1.8 mM KH_2_PO_4_) instead of drinking reverse osmosis water after the second renal surgery. This high phosphate loading with PBS in drinking water was described earlier [11]. Four months later, mice were sacrificed under isoflurane anesthesia followed by cervical dislocation and all efforts were made to alleviate suffering. When animals reached humane endpoints such as mobility loss, increased respiratory rate, and rapid weight loss of 20% within a few days, they were sacrificed. Two animals in nephrectomized BKO+PBS group met the humane endpoints and were sacrificed. Blood samples were collected for complete blood count analysis at the Faculty of Veterinary Science, Chulalongkorn University. Serum was separated by centrifugation for 15 min at 14,000 rpm and frozen at -80°C until blood chemistry analysis. Right femurs were frozen in liquid nitrogen, and kept at -80°C for RNA extraction and qPCR analysis. Left femurs were fixed in 70% alcohol for μCT and histomorphometry analysis. Left kidneys were fixed in 10% neutral buffered formalin for histological studies.

### μCT analysis

Bone microarchitecture measurements were performed using a desktop μCT35, (Scanco Medical, Basserdorf, Switzerland) according to recommended guidelines [12]. Cancellous and cortical bone microarchitecture was determined using a 7 μm isotropic voxel size. The x-ray source was operated at 70 kVp and 113 μA. Bone segmentation was evaluated using a threshold at 35 and 19% of the maximal gray scale values for cortical and cancellous bone, respectively. Cancellous bone was assessed in 300 transverse slices whereas 86 slices of cortical bone were evaluated. Bone parameters included bone volume per tissue volume (BV/TV, %), trabecular number (Tb.N, /mm), trabecular thickness (Tb.Th, mm), trabecular separation (Tb.Sp, mm), connectivity density (ConnD, /mm^3^), structure model index (SMI, -), cross-sectional volume (mm^3^), cortical volume (mm^3^), marrow volume (mm^3^), cortical thickness (mm), and bone mineral density (mgHA/cm^3^).

### Bone histomorphometry

Mice were subcutaneously injected with 20 mg/kg body weight calcein (Sigma, St. Louis, MO, USA) 9 and 2 days before sacrifice. Femurs were dehydrated in gradient acetone and processed undecalcified in methyl methacrylate. Sample sections were cut at 5 μm thickness using a Leica RM2255 motorized microtome and unstained for dynamic measurements as previously described [13]. Consecutive sections were stained with toluidine blue for the measurement of static bone parameters. Measurements were evaluated in the distal metaphysis of femurs at 400 μm below the growth plate using a semi-automatic image analysis system (Osteometrics Inc., Decatur, GA). All parameters were expressed according to the standardized nomenclature [14].

### qPCR analysis

Total RNA was extracted from right femurs using Trizol reagent (Invitrogen, Carlsbad, CA, USA), a mixture of guanidine thiocyanate and phenol in a monophase solution following the manufacturer’s procedure. RNA samples were cleaned up using RNeasy Mini kit (Qiagen, Germantown, MD, USA), and RNA yields were determined by spectrophotometry at 260 nm using a NanoDrop 1000 (Thermo Fisher Scientific, CA, USA). RNA integrity was assessed using a 1% denaturing agarose gel electrophoresis stained with ethidium bromide and visualized on a UV transilluminator (Syngene GeneGenius, Schonwalde-Glien, Germany). cDNA was synthesized from 1 μg of total RNA using SuperScript VILO cDNA synthesis kit (Invitrogen, Carlsbad, CA, USA). The qPCR analysis was performed with the Luna Universal qPCR Master Mix (New England Biolabs, Ipswich, MA, USA) at 60°C for 40 cycles using CFX96^TM^ Optics Module (Bio-Rad, Hercules, CA, USA). qPCR data were normalized to *Gapdh* as an internal control. The oligonucleotide primer sequences are described in supplementary S1 Table.

### Renal histology

The left kidney was dehydrated, embedded in paraffin, and cut at 5 μm thick sections. The renal interstitial fibrosis and tubular atrophy (IFTA) score, a primary end points for assessing kidney injury and fibrosis was estimated at 20X magnification on Masson’s Trichrome stained sections using 10 randomly selected fields. A semi-quantitative scale was applied: 0, area of damage <5%; 1, area of damage 5–10%; 2, area of damage 11–25%; 3, damage involving 26–50%; and 4, damage involving >50% of the kidney section.

### Serum chemistries

Serum urea nitrogen, creatinine, phosphorus and calcium levels were analyzed as per the manufacturer’s instructions (Standbio Laboratory, Boerne, TX). Serum biochemistries were measured using ELISA kits for erythropoietin (R&D systems, Minneapolis, MN), FGF23 (Quidel, San Diego, CA), and PTH levels (Quidel, San Diego, CA). Serum FGF23 ELISA kit detected both intact and c-terminal fragments of FGF23.

### Statistical analysis

Multiple comparisons were analyzed using two-way ANOVA followed by least significant difference (LSD) *post hoc test*. Data are expressed as violin plots, synergistical combining the density trace and summary statistics relevant to boxplots. A *p*-value < 0.05 was considered statistically significant.

## Results

### BKO mice displayed hematological characteristics of anemia

First, the thalassemic phenotype of BKO mice under standard and nephrectomy conditions was determined. Hematological indices including RBC, hemoglobin (Hb), hematocrit (Hct), mean cell volume (MCV), mean cell hemoglobin (MCH), and mean corpuscular hemoglobin concentration (MCHC) were reduced, whereas red cell distribution width-coefficient of variation (RDW-CV) and red cell distribution width-standard deviation (RDW-SD) were increased in sham BKO mice compared to sham WT controls (S2 Table). BKO mice were anemic and had red blood cell morphologic changes, including microcytosis, hypochromia, anisocytosis (varying size), poikilocytosis (varying shape), and nucleated RBC. Nephrectomy resulted in anemia indicated by decreased RBC, Hb, and Hct levels in WT and diminished RBC in BKO mice. PBS in drinking water did not have any effect on RBC, Hb, and Hct levels in WT compared to nephrectomized WT. However, Hct, and MCV were decreased in nephrectomized BKO+PBS mice compared to nephrectomized BKO mice.

### High phosphate intake enhances renal fibrosis and serum phosphorus levels in nephrectomized BKO mice

**Fig 1:**
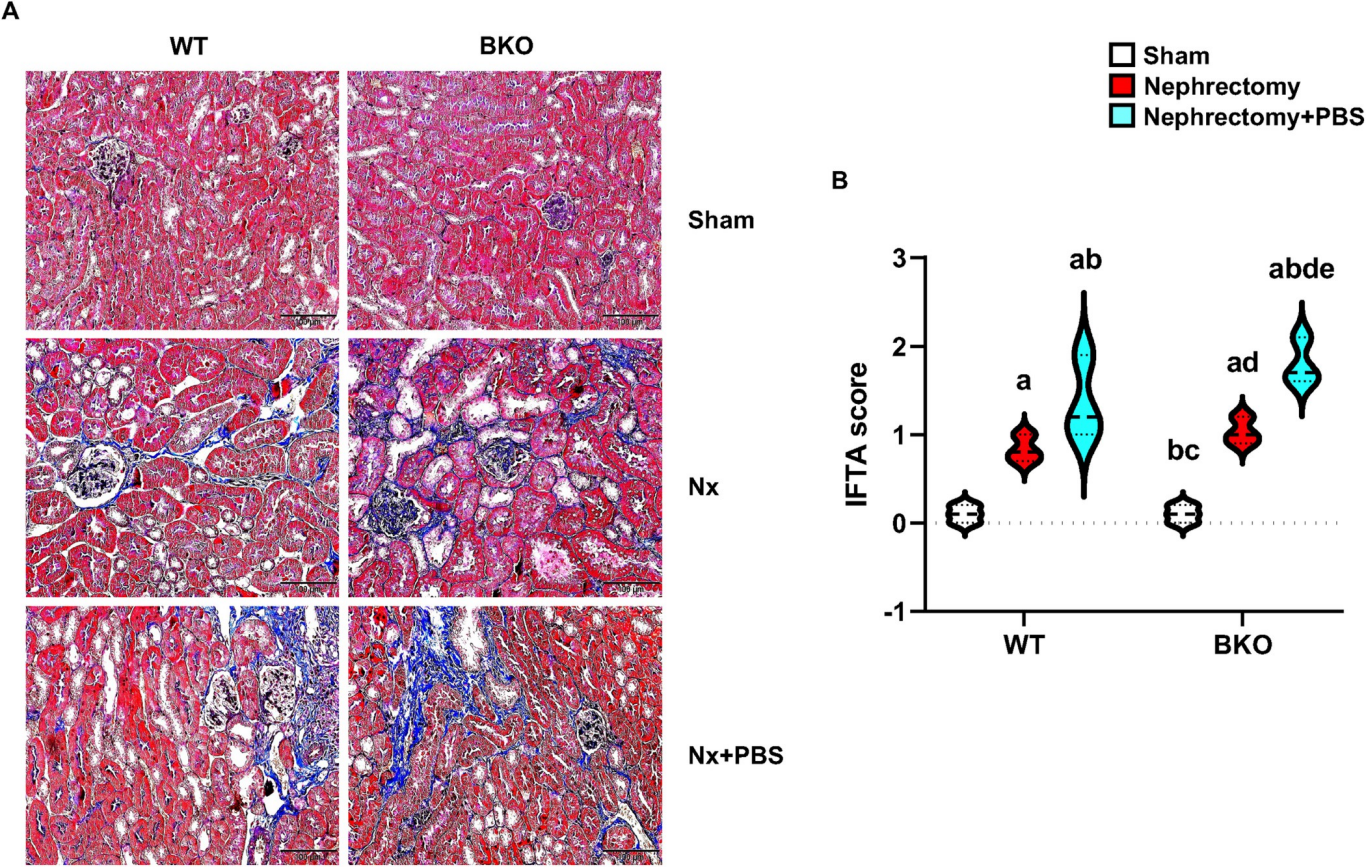
High phosphate enhances renal fibrosis in nephrectomized WT and BKO mice. (A) Kidneys stained with Masson’s Trichrome (n = 3 per group) from sham WT, nephrectomized WT, nephrectomized WT+PBS, sham BKO, nephrectomized BKO, and nephrectomized BKO+PBS mice. Blue color indicates fibrosis with an accumulation of collagen. (B) Violin plot showing IFTA scores in the kidneys. ^a^*p* < 0.05 versus sham WT, ^b^*p* < 0.05 versus nephrectomized WT, ^c^*p* < 0.05 versus nephrectomized WT+PBS, ^d^*p* < 0.05 versus sham BKO, and ^e^*p* < 0.05 versus nephrectomized BKO. Scale bar: 100 μm.

Renal fibrosis is a non-reversible scarring, one of the best histological markers of progressive kidney disease. Sham WT and BKO mice did not have renal fibrosis, whereas nephrectomized WT and BKO exhibited tubulointerstitial fibrosis and glomerulosclerosis within the renal tissues. In particular, the incidence of fibrosis was highest in nephrectomized WT and nephrectomized BKO mice fed PBS (Fig 1A). Similarly, IFTA scores were increased in nephrectomized WT and BKO mice, and the scores were much higher in either nephrectomized WT+PBS or nephrectomized BKO+PBS (Fig 1B).

**Fig 2:**
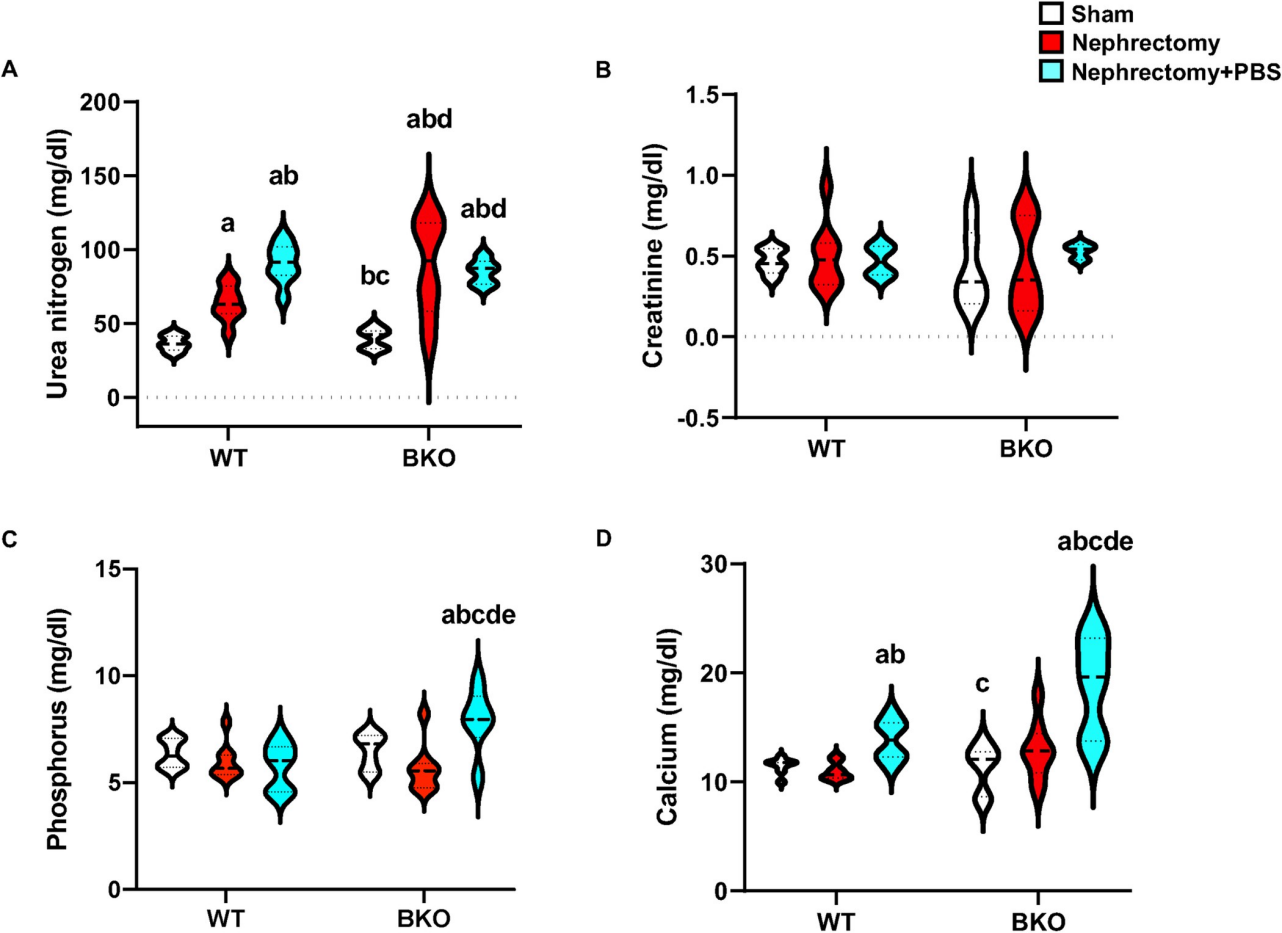
Nephrectomy increases serum urea nitrogen and high phosphate enhances serum phosphorus and calcium in nephrectomized BKO mice. (A) Serum urea nitrogen from sham WT (n = 8), nephrectomized WT (n = 8), nephrectomized WT+PBS (n = 8), sham BKO (n = 7), nephrectomized BKO (n = 8), and nephrectomized BKO+PBS mice (n = 5). (B) Serum creatinine from sham WT (n = 5), nephrectomized WT (n = 8), nephrectomized WT+PBS (n = 4), sham BKO (n = 6), nephrectomized BKO (n = 8), and nephrectomized BKO+PBS mice (n = 3). (C) Serum phosphorus from sham WT (n = 8), nephrectomized WT (n = 8), nephrectomized WT+PBS (n = 8), sham BKO (n = 7), nephrectomized BKO (n = 8), and nephrectomized BKO+PBS mice (n = 6). (D) Serum calcium from sham WT (n = 8), nephrectomized WT (n = 8), nephrectomized WT+PBS (n = 8), sham BKO (n = 7), nephrectomized BKO (n = 8), and nephrectomized BKO+PBS mice (n = 6). ^a^*p* < 0.05 versus sham WT, ^b^*p* < 0.05 versus nephrectomized WT, ^c^*p* < 0.05 versus nephrectomized WT+PBS, ^d^*p* < 0.05 versus sham BKO, and ^e^*p* < 0.05 versus nephrectomized BKO.

To evaluate the renal excretory function, serum levels of urea nitrogen were analyzed (Fig 2). Sham BKO mice did not affect serum urea nitrogen levels. In contrast, there was approximately a 2- and 2.4-fold increase in serum urea nitrogen levels in nephrectomized WT and BKO mice as compared to their corresponding controls (Fig 2A). PBS intake caused the induction of serum urea nitrogen levels in nephrectomized WT but not BKO mice. Serum creatinine levels were similar in all groups (Fig 2B). Serum phosphorus levels were not changed in sham BKO mice, nephrectomized WT or nephrectomized BKO mice. Interestingly, serum phosphorus levels dramatically increased in nephrectomized BKO+PBS mice (Fig 2C), indicating an interaction between BKO and nephrectomy-induced susceptibility to PBS-induced hyperphosphatemia. Nephrectomy did not have any effect on serum calcium levels in WT and BKO mice (Fig 2D). However, PBS intake increased serum calcium levels in both nephrectomized WT and BKO mice. Serum calcium levels were highest in nephrectomized BKO+PBS mice.

### High phosphate intake induces cancellous and cortical bone loss in nephrectomized BKO mice

**Fig 3:**
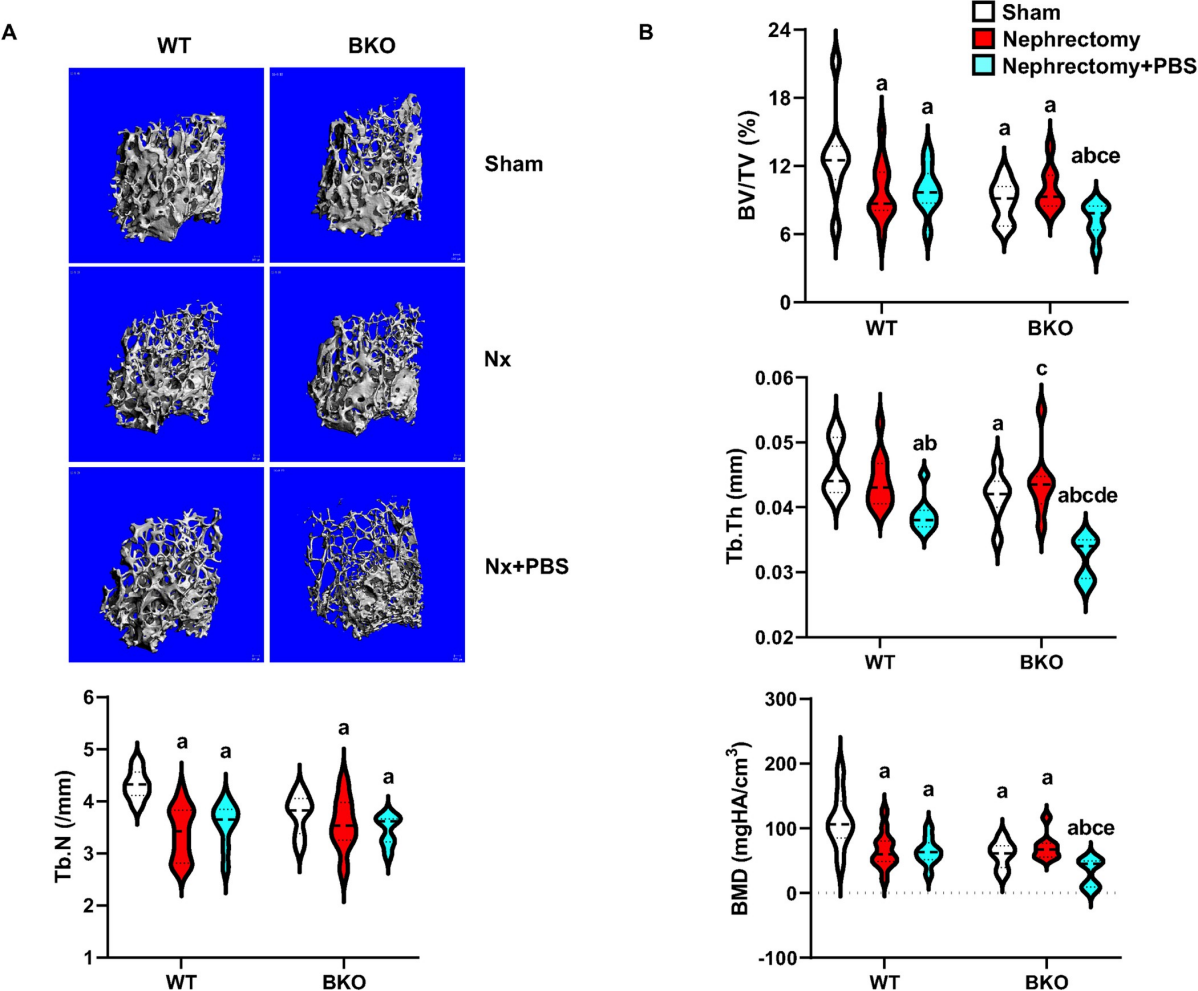
High phosphate induces cancellous bone loss in nephrectomized BKO mice. (A) Representative μCT images of cancellous bone. (B) μCT analysis of cancellous bone as violin plots. ^a^*p* < 0.05 versus sham WT, ^b^*p* < 0.05 versus nephrectomized WT, ^c^*p* < 0.05 versus nephrectomized WT+PBS, ^d^*p* < 0.05 versus sham BKO, and ^e^*p* < 0.05 versus nephrectomized BKO.

**Fig 4:**
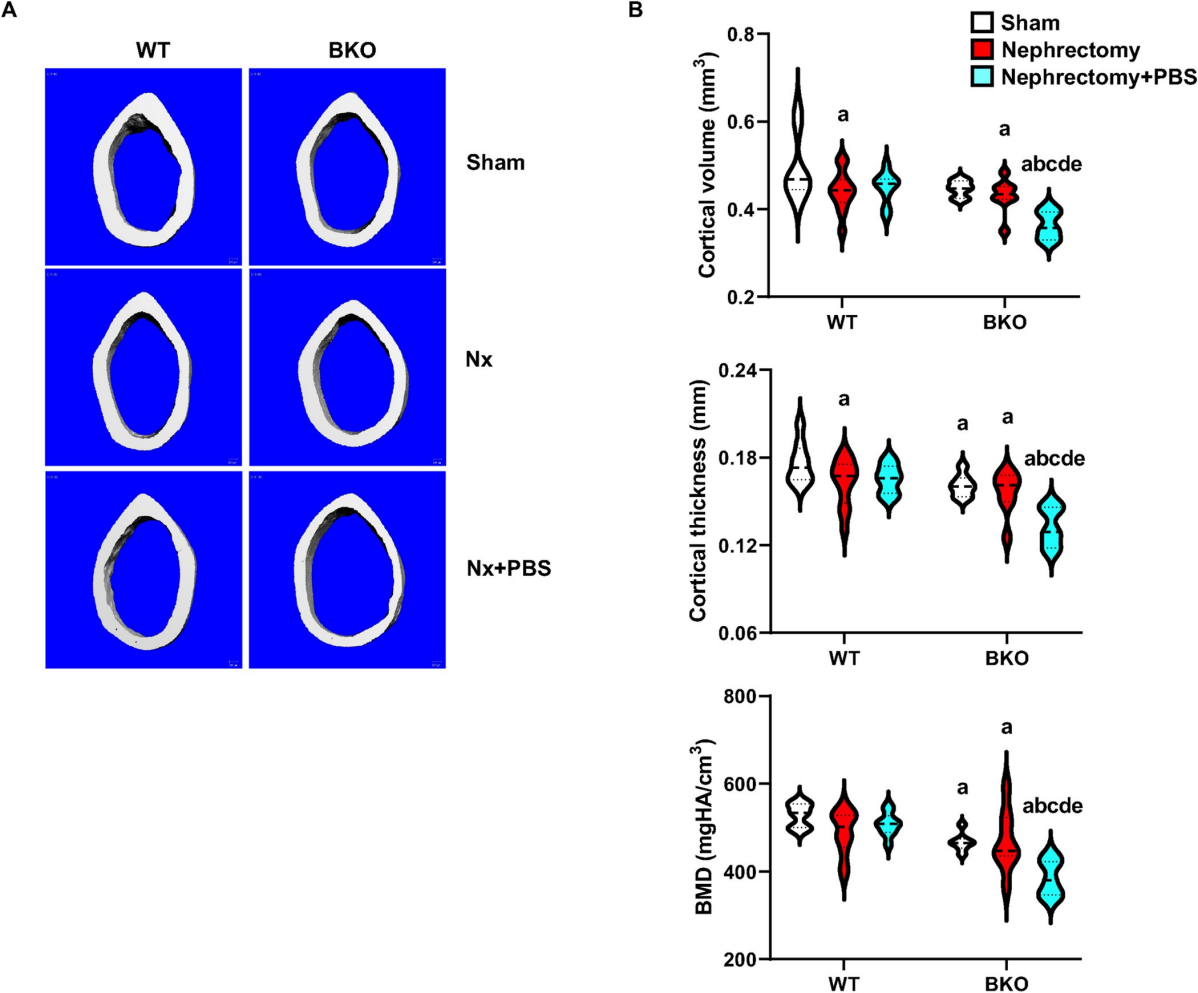
High phosphate increases cortical bone loss in nephrectomized BKO mice. (A) Representative μCT images of cortical bone. (B) μCT analysis of cortical bone as violin plots. ^a^*p* < 0.05 versus sham WT, ^b^*p* < 0.05 versus nephrectomized WT, ^c^*p* < 0.05 versus nephrectomized WT+PBS, ^d^*p* < 0.05 versus sham BKO, and ^e^*p* < 0.05 versus nephrectomized BKO.

To investigate whether the PBS intake induced femoral cancellous and cortical bone loss in thalassemic mice, μCT analysis was performed. Sham BKO mice had an osteopenic phenotype due to a significant reduction in cancellous bone volume, trabecular thickness, cortical thickness, and bone mineral density of cancellous and cortical bone compared to sham WT (Figs 3 and 4 and S3 Table). Nephrectomy decreased cancellous bone volume, trabecular number and bone mineral density in WT (Fig 3A and 3B). Cortical bone volume and cortical thickness were also decreased by nephrectomy in WT (Fig 4A and 4B). However, BKO-induced femoral bone loss was not altered by nephrectomy. PBS intake did not impact femoral cancellous and cortical bone in nephrectomized WT, except that trabecular thickness was decreased (Fig 3B). However, PBS obviously induced femoral cancellous and cortical bone loss in BKO mice. Cancellous bone volume, trabecular thickness, and bone mineral density were decreased (Fig 3B). For cortical bone, cortical bone volume, cortical thickness, and bone mineral density were reduced (Fig 4B). Thus, BKO and nephrectomy act together to synergistically produce a susceptibility to phosphate-induced bone loss. Two-way ANOVA showed an interaction between BKO and nephrectomy on cancellous bone volume and BMD and interaction between BKO and PBS on cortical bone volume, cortical thickness and BMD (S5 Table).

**Fig 5:**
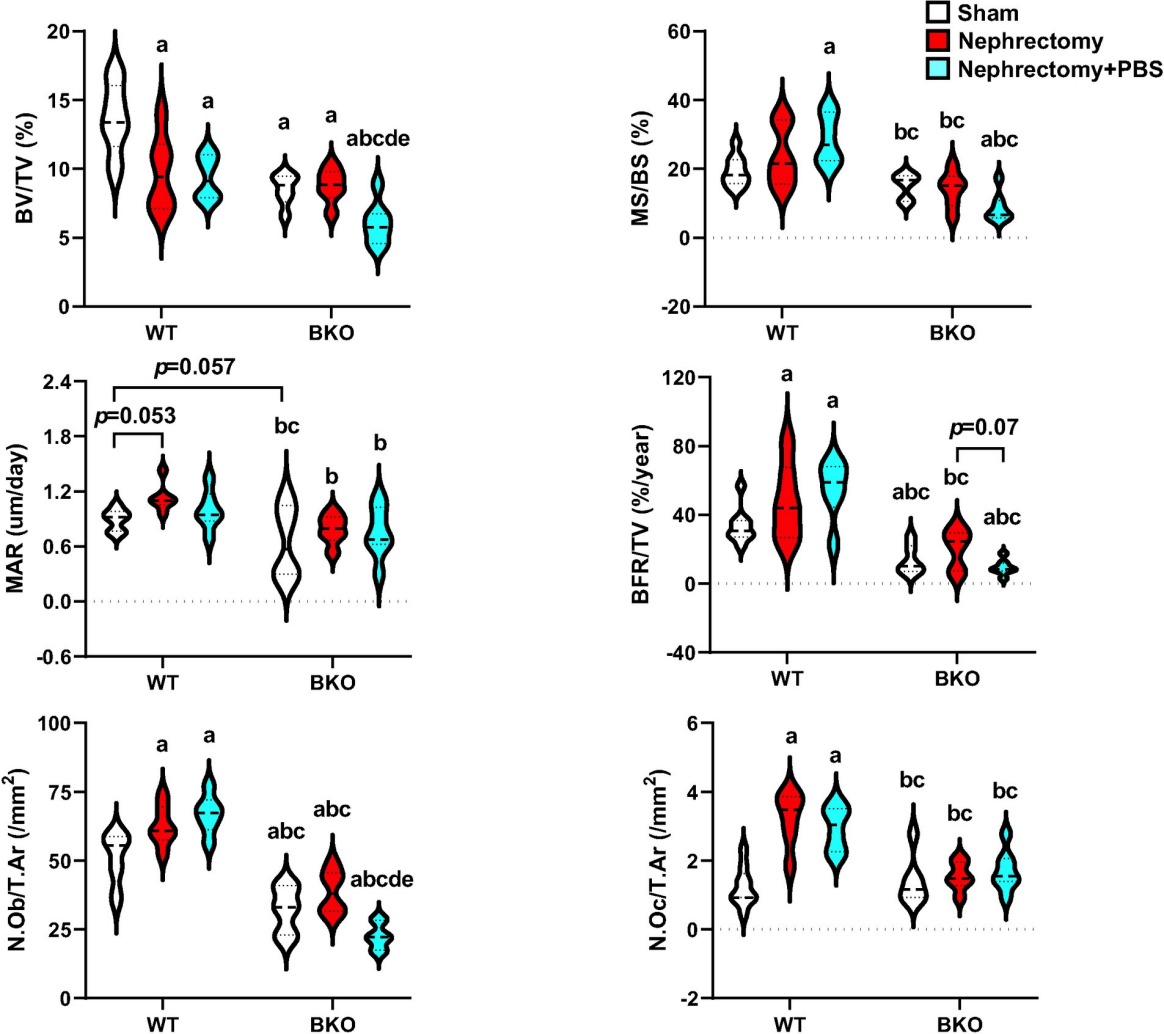
Bone histomorphometric analysis of femurs in BKO mice and WT controls after nephrectomy and PBS exposure. ^a^*p* < 0.05 versus sham WT, ^b^*p* < 0.05 versus nephrectomized WT, ^c^*p* < 0.05 versus nephrectomized WT+PBS, ^d^*p* < 0.05 versus sham BKO, and ^e^*p* < 0.05 versus nephrectomized BKO.

Furthermore, bone histomorphometric analysis confirmed that sham BKO mice had lower cancellous bone volume than WT controls due to a significant reduction in bone formation rate, and osteoblast number, and a slight decrease in mineral apposition rate (MAR, *p* = 0.057), whereas osteoclast number was not changed (Fig 5 and S4 Table). Nephrectomy decreased cancellous bone volume due to increased bone formation and bone resorption suggesting increased bone turnover in WT but not BKO mice. While mineralizing surface (MS/BS) was not affected, mineral apposition rate was slightly increased, leading to an increase in bone formation rate. Osteoblast number and osteoclast number were both increased (Fig 5). PBS intake reduced cancellous bone volume in nephrectomized BKO but not nephrectomized WT mice. Cancellous bone loss resulted from dramatically decreased bone formation rate and osteoblast number when compared to nephrectomized WT exposed to PBS. Therefore, μCT and histomorphometric analysis indicated that PBS intake provoked femoral bone loss during the development of CKD in thalassemic mice.

### The combined transcriptional impact of thalassemia and nephrectomy on skeletal phosphate responses

**Fig 6:**
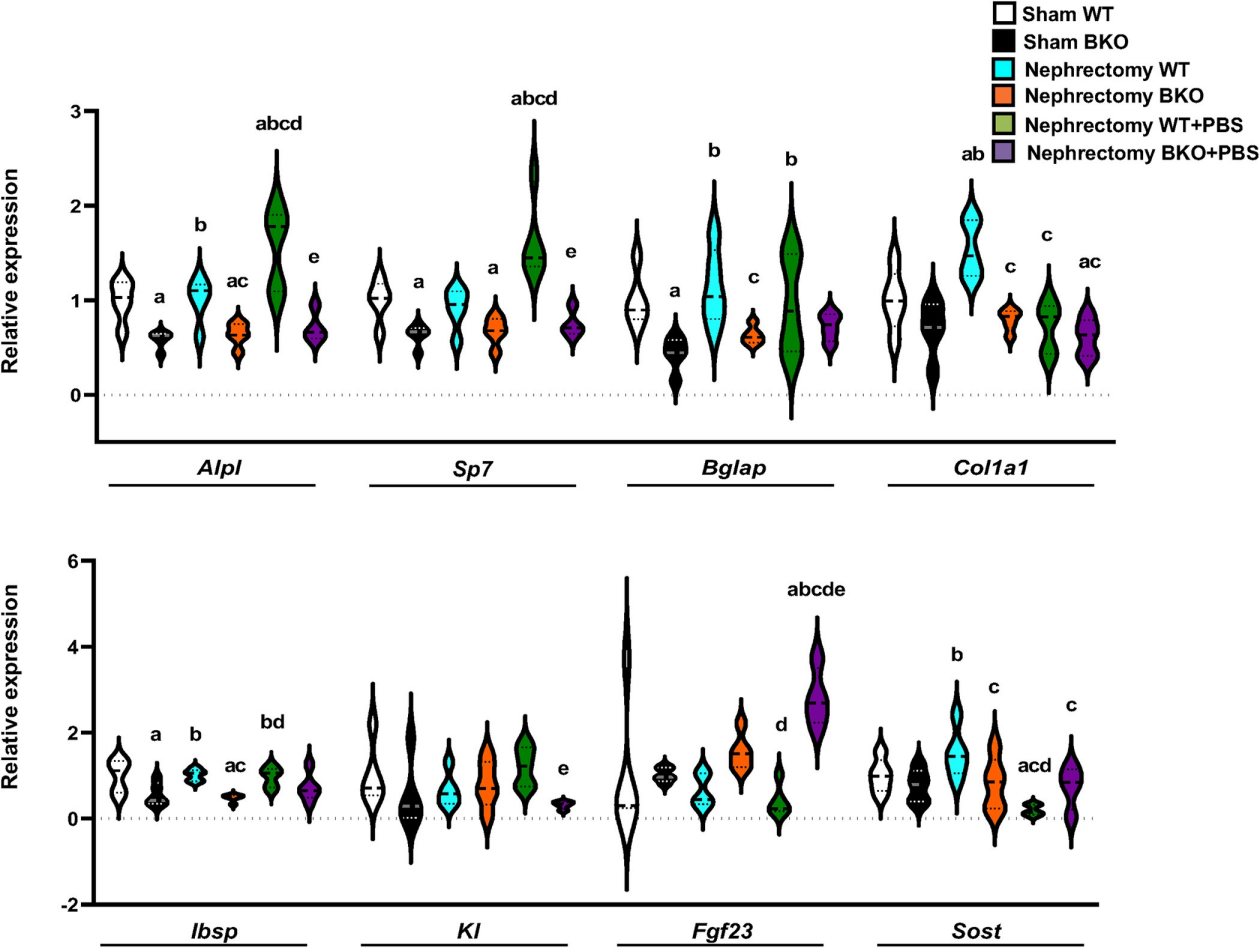
Nephrectomy of WT with or without PBS exposure upregulates osteoblast-related gene expression in femurs. Violin plots for qPCR analysis of osteoblast-related genes (n = 4–5 per group). ^a^*p* < 0.05 versus sham WT, ^b^*p* < 0.05 versus sham BKO, ^c^*p* < 0.05 versus nephrectomized WT, ^d^*p* < 0.05 versus nephrectomized BKO, and ^e^*p* < 0.05 versus nephrectomized WT+PBS.

**Fig 7:**
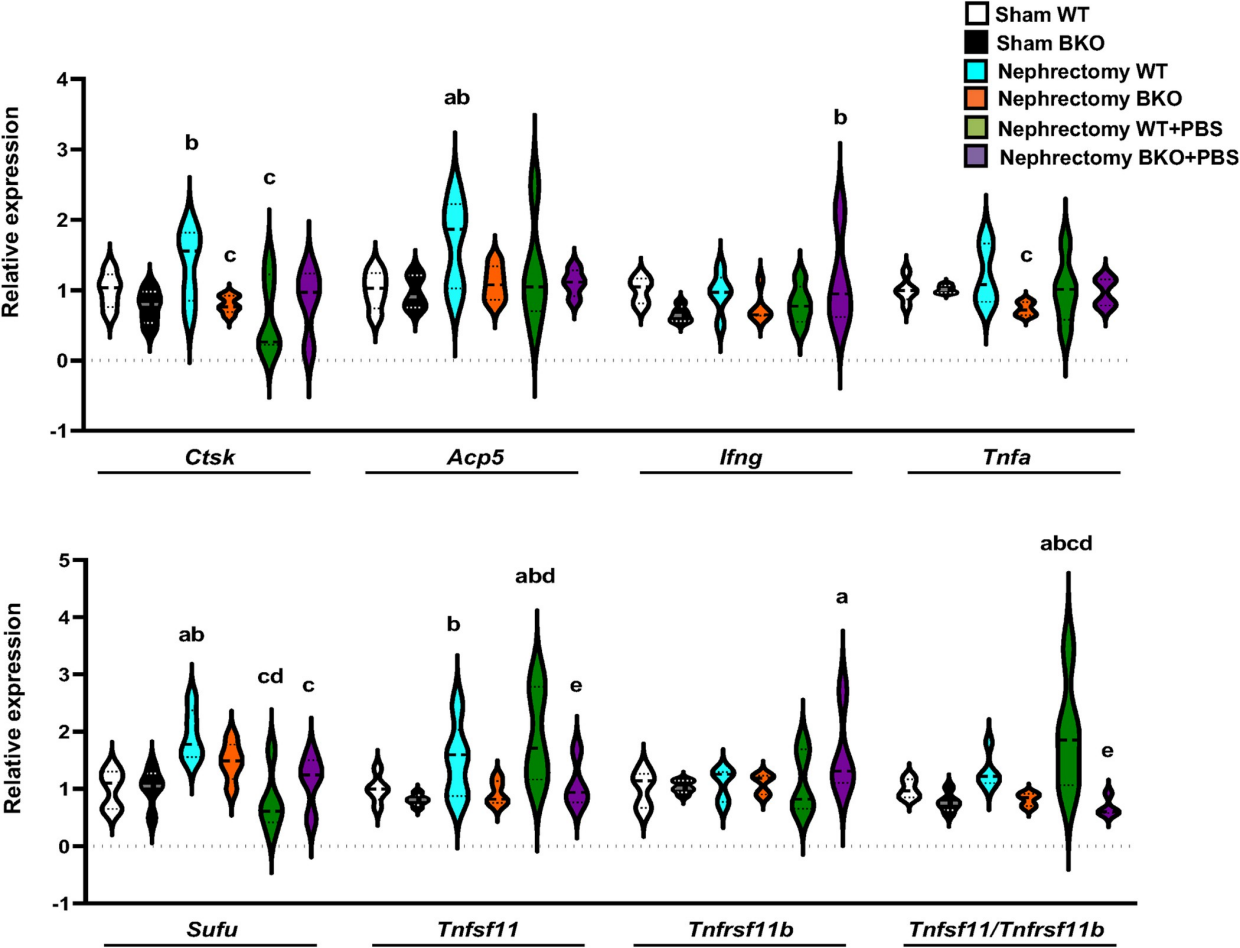
Nephrectomy in WT with or without PBS exposure upregulates osteoclast-related gene expression in femurs. Violin plots for qPCR analysis of osteoclast-related genes (n = 4–5 per group). ^a^*p* < 0.05 versus sham WT, ^b^*p* < 0.05 versus sham BKO, ^c^*p* < 0.05 versus nephrectomized WT, ^d^*p* < 0.05 versus nephrectomized BKO, and ^e^*p* < 0.05 versus nephrectomized WT+PBS.

To investigate the alteration of transcriptional profile related to bone homeostasis, the expression of osteoblast and osteoclast-related genes was performed by qPCR analysis (Fig 6). qPCR results showed a significant downregulation in osteoblast-related gene expression, including *Alpl*, *Sp7*, *Bglap*, and *Ibsp* (Fig 6), whereas there was no change in osteoclast-related gene expression in sham BKO mice (Fig 7). Nephrectomy led to an approximately 2-fold increase in the expression levels of type I collagen α1 (*Col1α1*), *Acp5*, and *Sufu* in WT control. *Alpl*, *Bglap*, *Col1α1*, *Ibsp*, *Ctsk and Tnfa* were decreased in nephrectomized BKO mice compared to nephrectomized WT. However, the expression levels of genes related to osteoblastogenesis and osteoclastogenesis were not altered in nephrectomized BKO when compared to sham BKO. The mRNA levels of *Alpl* and *Sp7* were significantly increased, whereas *Col1α1* and *Sost* expression were reduced in nephrectomized WT receiving PBS. PBS intake downregulated the expression of *Ctsk* and *Sufu* but upregulated the *Tnfsf11/Tnfrsf11b* ratio in nephrectomized WT controls. PBS intake did not alter osteoblast and osteoclast gene expressions in nephrectomized BKO mice compared to nephrectomized BKO mice. However, PBS-exposed nephrectomized BKO mice had decreased *Alpl*, *Sp7*, *Kl*, *Tnfsf11*, and *Tnfsf11/Tnfrsf11b* compared to PBS-exposed nephrectomized WT. Interestingly, the mRNA levels of *Fgf23*, a bone-derived hormone produced by osteocytes and osteoblasts in response to hyperphosphatemia, were remarkably increased by approximately 7-fold in nephrectomized BKO mice compared to nephrectomized WT following PBS intake (Fig 6). Therefore, *Fgf23* is probably a key regulator of mineral metabolism in CKD thalassemic mice during hyperphosphatemia.

### PBS increases serum erythropoietin, FGF23, and PTH levels in nephrectomized BKO mice

**Fig 8:**
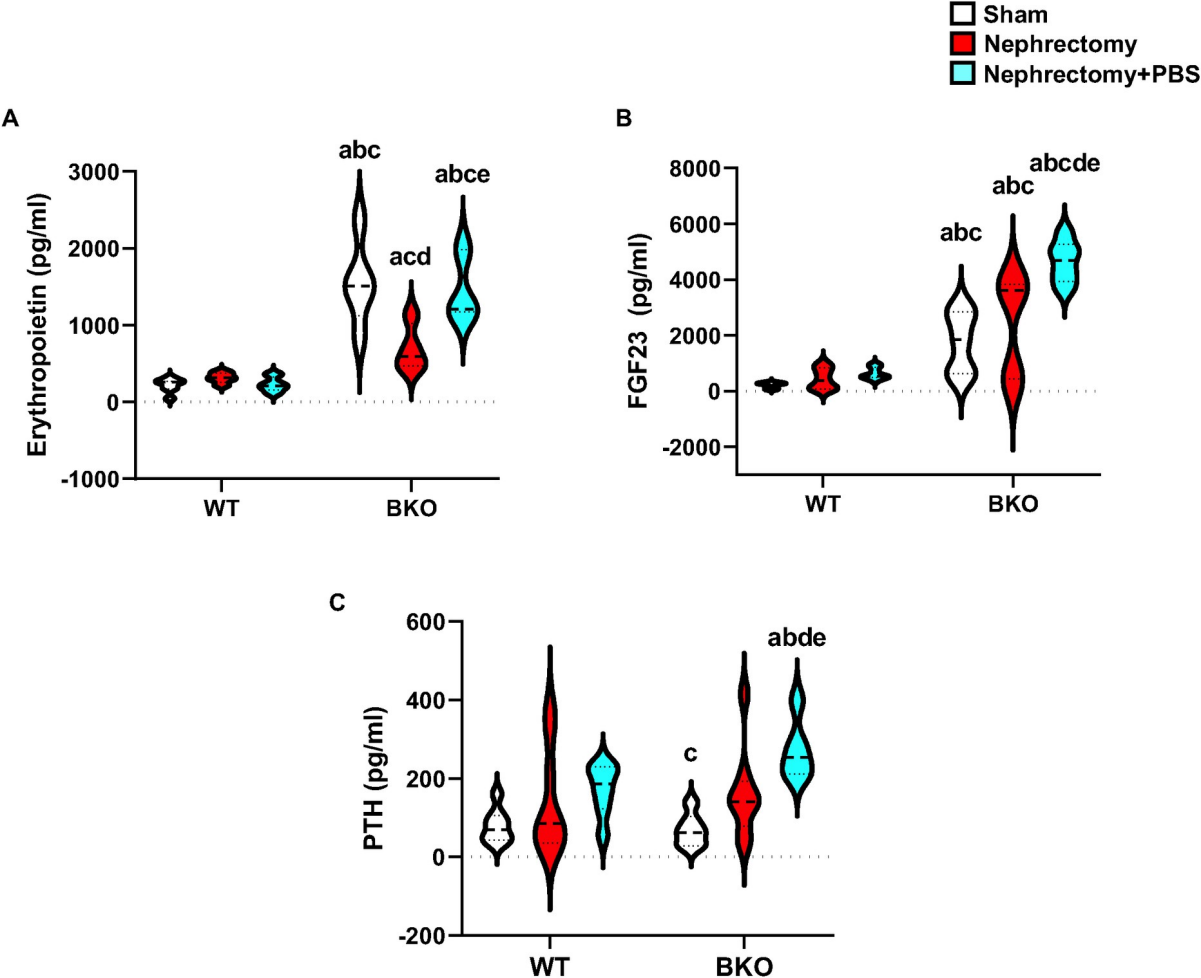
High phosphate increases serum erythropoietin, FGF23, and PTH levels in nephrectomized BKO mice. (A) Serum erythropoietin from sham WT (n = 7), nephrectomized WT (n = 5), nephrectomized WT+PBS (n = 8), sham BKO (n = 5), nephrectomized BKO (n = 4), and nephrectomized BKO+PBS mice (n = 3). (B) Serum FGF23 from sham WT (n = 7), nephrectomized WT (n = 7), nephrectomized WT+PBS (n = 8), sham BKO (n = 5), nephrectomized BKO (n = 5), and nephrectomized BKO+PBS mice (n = 6). (C) Serum PTH from sham WT (n = 8), nephrectomized WT (n = 9), nephrectomized WT+PBS (n = 7), sham BKO (n = 6), nephrectomized BKO (n = 8), and nephrectomized BKO+PBS mice (n = 5). ^a^*p* < 0.05 versus sham WT, ^b^*p* < 0.05 versus nephrectomized WT, ^c^*p* < 0.05 versus nephrectomized WT+PBS, ^d^*p* < 0.05 versus sham BKO, and ^e^*p* < 0.05 versus nephrectomized BKO.

Erythropoietin, an endocrine hormone predominantly released by the kidney in response to hypoxemia, increases red blood cell mass by promoting the survival of erythrocytic progenitors. Serum levels of erythropoietin were not altered by nephrectomy or nephrectomy+PBS in WT controls (Fig 8A). Interestingly, erythropoietin levels were remarkably increased by 7.1-fold in sham BKO compared to sham WT. However, these elevated levels were abated in BKO mice following nephrectomy. On the other hand, PBS intake increased the production of serum erythropoietin levels in nephrectomized BKO mice comparable to those in nephrectomized BKO mice. These results suggested that erythropoietin not only served as an essential hormone responsible for erythropoiesis in thalassemic mice but also indicate an expected link between erythropoietin production and phosphate metabolism. Two-way ANOVA indicated an interaction between BKO and nephrectomy, and between BKO and PBS on erythropoietin levels (S5 Table).

Both FGF23 and PTH are phosphaturic hormones and important regulators of mineral homeostasis. Nephrectomy or nephrectomy+PBS did not affect the serum FGF23 and PTH levels in WT controls (Fig 8B and 8C). Sham BKO and nephrectomized BKO both displayed markedly increased FGF23 but not PTH levels when compared to their corresponding WT controls. Although serum levels of FGF23 and PTH were not altered in BKO following nephrectomy compared to sham BKO, serum FGF23 and PTH levels were dramatically increased by 1.9- and 1.7-fold in nephrectomized BKO+PBS mice when compared to nephrectomized BKO, respectively. Nephrectomized BKO+PBS mice had increased serum levels of FGF23 and PTH by 7.4- and 1.6-fold compared to nephrectomized WT+PBS. These data supported that serum FGF23 and PTH were responsible for the regulation of phosphorus homeostasis in thalassemic mice following nephrectomy.

## Discussion

Patients with either β-thalassemia or CKD generally suffer from severe osteoporosis and bone fragility due to their low bone mineral density [15]. Hyperphosphatemia is commonly observed in late-stage CKD and is a driver of mineral bone disorder (CKD-MBD) [16]. Understanding the pathophysiology of CKD-induced skeletal disorder in thalassemia may provide benefits to discover the therapeutic targets for skeletal deformity. The present study uncovered an unexpected interaction among CKD as modeled by 5/6^th^ nephrectomy, thalassemic state and phosphate-induced skeletal disorder. Herein, only BKO mice were used because homozygous *β-globin* knockout mice are embryonic lethal. Four months after nephrectomy, all nephrectomized mice had renal tubulointerstitial fibrosis, as well as increased serum urea nitrogen levels, confirming renal impairment. BKO mice were severely anemic and had cancellous and cortical bone loss. The microstructural defect of BKO bone was due to reduced bone formation. Nephrectomy induced cancellous and cortical bone loss and increased both osteoblast and osteoclast number in WT controls, indicating increased bone turnover. However, the increased bone loss following nephrectomy did not occur in BKO mice possibly due to low level of erythropoietin. Interestingly, high phosphate intake enhanced renal fibrosis in both nephrectomized WT and BKO mice, indicating end-state CKD. The cancellous and cortical bone volume, cortical thickness, and bone mineral density were worsened in nephrectomized BKO+PBS mice, along with marked decreases in bone formation rate, and osteoblast number. Although PBS increased serum urea nitrogen levels in nephrectomized WT but not nephrectomized BKO, serum phosphorus and calcium were obviously elevated in nephrectomized BKO+PBS mice. In addition, PBS induced increases in serum levels of FGF23 and PTH in nephrectomized BKO. The evaluation of serum biochemistries and hormones indicated that high phosphate intake induces hyperphosphatemia, hypercalcemia, and hyperparathyroidism in thalassemic mice following renal insufficiency. Furthermore, highly increased erythropoietin levels in BKO mice were reduced by nephrectomy, whereas this reduction was compromised by PBS. In other words, high phosphate intake stimulated the production of erythropoietin in thalassemic mice but not WT controls following nephrectomy.

The hematological characteristics of β-thalassemia include microcytosis (reduced RBC volume), and hypochromia (reduced RBC and Hb content), associated with low MCV and MCH [17]. Low MCHC was also seen reflecting a reduction of the concentration of Hb in RBCs even when accounting for differences in volume. RDW, variation in RBC size (anisocytosis), tends to be high in patients with thalassemia [18]. In the present study, male BKO mice exhibited similar hematological characteristics to thalassemia patients. RBC, Hb, Hct, MCV, and MCH were decreased whereas RDW-CV and RDW-SD were increased in BKO mouse model. Moreover, 5/6^th^ nephrectomy induced anemia in both WT and BKO mice. The kidney is the main source of erythropoietin secretion, a hormone that plays a key role in the production of RBCs. Thus, anemia in CKD is commonly attributed to erythropoietin insufficiency [19]. Although nephrectomy induced anemia in WT, serum erythropoietin levels did not change. Chronic anemia in thalassemia markedly stimulates the erythropoietin production, however, elevated erythropoiesis is ineffective because erythroid progenitor precursors fail to mature, and die in the process of becoming erythrocytes. We found high phosphate intake induced more severe anemia in nephrectomized BKO but not nephrectomized WT. Kovesdy *et al*. suggested that high serum phosphorus led to higher uremic toxin production, inhibiting erythropoiesis [20].

Thalassemia-induced bone loss is caused by hypogonadism, GH deficiency, iron overload, chronic anemia and progressive bone marrow expansion due to ineffective erythropoiesis. In β-thalassemia, erythropoietin is dramatically increased in response to anemia and hypoxia with subsequent erythroid expansion in bone marrow resulting in bone deformity [21]. However, the mechanisms by which erythropoietin induces bone loss have not been clearly clarified. In transgenic mice overexpressing erythropoietin, high erythropoietin levels provoked erythropoiesis accompanied by cancellous bone loss, decreased bone formation, and increased bone resorption [22]. We determined that very high erythropoietin production in thalassemic mice was associated with femoral bone loss. Since erythropoietin was mainly produced in the kidney, the low erythropoietin levels resulting from CKD prevented nephrectomy-induced femoral bone loss in BKO mice when compared to sham BKO mice. Erythropoietin levels that turned to be high possibly due to severe anemia in nephrectomized BKO drinking PBS induced greater degrees of bone loss.

Although it was previously revealed that the abnormalities of renal function resulting in renal failure are common in thalassemic patients [23], serum albumin, calcium, phosphorus, creatinine, and BUN are commonly normal in these patients [24]. Similarly, our BKO mice had normal renal function at least as assessed by normal serum urea nitrogen, creatinine, calcium and phosphorus levels. Although we could not observe any change in serum creatinine levels after nephrectomy, it did not mean that nephrectomy did not induce renal failure. It has been reported that some patients with a normal range of creatinine levels may have a substantial reduction in kidney function [25]. BUN concentration is an easily measurable and widely available marker of renal function and the BUN levels inversely correlate with the GFR. As expected, nephrectomized WT and BKO mice with or without PBS had high levels of serum urea nitrogen levels, indicating renal dysfunction. Our study was consistent with other established CKD mouse and rat models which all share high serum BUN levels [16,26]. Although PBS increased serum urea nitrogen levels in nephrectomized WT, serum urea nitrogen levels that was already high in nephrectomized BKO did not alter following PBS loading. In high phosphate loading, serum calcium levels were increased in both nephrectomized WT and BKO mice possibly due to slightly increased PTH levels in nephrectomized WT and significantly increased PTH in nephrectomized BKO mice. PTH induces 1,25(OH)_2_D_3_ synthesis and promotes calcium release from bone, and enhances intestinal calcium absorption.

Patients with β-thalassemia have low bone mass, bone pain, deformities, and pathological fractures. Several factors contribute to the reduction in bone mass observed, including genetic factors, iron toxicity, hormonal deficiency, bone marrow expansion, and increased bone turnover [27,28]. In our study, BKO mice had cancellous and cortical bone loss in the femur by decreased osteoblast number, bone formation rate, and downregulation of osteoblast-related genes, including *Alpl*, *Sp7*, *Bglap*, and *Ibsp*, without altering osteoclast number. Our findings were consistent with previous reports [29]. Thus, femoral bone loss in the BKO mice was mainly due to impaired osteoblast function without accelerated osteoclastic bone resorption. According to KDIGO guidelines, abnormalities in bone turnover, bone mineralization, and bone mass in CKD are systemic complications of CKD-MBD [30]. Studies in CKD patients demonstrated that higher biochemical markers of bone turnover were associated with lower cortical BMD [31] and cancellous BMD at the lumbar spine, total hip, and femoral neck [32]. Interestingly, cancellous and cortical bone loss were markedly increased in nephrectomized BKO drinking PBS without changes in osteoblast- and osteoclast-related gene, whereas osteoblast number was significantly decreased concomitant with slight decreases in mineralizing surface and bone formation rate compared to nephrectomized BKO mice. However, *Fgf23* expression was highly elevated. FGF23, a phosphaturic hormone, is induced by hyperphosphatemia, and plays a crucial role in bone mineralization. Therefore, endocrine-stimulating factors in response to hyperphosphatemia may contribute to bone loss in nephrectomized BKO+PBS.

FGF23 suppresses erythropoietin production whereas erythropoietin stimulates FGF23. A recombinant human erythropoietin activates serum FGF23 in mice with normal kidney function and acute kidney injury [33,34]. β-thalassemia intermedia mice (*Hbb*^*th3/+*^ mice) have been reported to have high serum FGF23 [35]. Similarly, our study indicated that high circulating FGF23 and erythropoietin levels were observed in sham BKO, nephrectomized BKO, and nephrectomized BKO+PBS mice. FGF23 increases early in CKD to prevent hyperphosphatemia, which in turn enhances renal phosphate excretion and represses renal phosphate reabsorption. FGF23 inhibits 1,25(OH)_2_D_3_ production limiting intestinal phosphate absorption and stimulates PTH secretion through its co-receptor, Klotho. The levels of FGF23 and PTH increase progressively and subsequently drive secondary hyperparathyroidism and CKD-MBD [36]. Transgenic mice overexpressing *Fgf23* display secondary hyperparathyroidism [37]. Nephrectomized BKO mice exposed to high phosphate loads had dramatically decreased cancellous bone volume due to decreased osteoblast number without alteration in bone resorption. It has been shown that the elevated FGF23 production promoted mineralization defects leading to rickets and osteomalacia [38], contributing to bone loss by inhibiting the osteoblastic Wnt signaling pathway [39]. In addition, high PTH levels were associated with abnormal cortical and cancellous bone density [32], and higher cortical porosity [40]. A longitudinal study in CKD patients has shown that hyperparathyroidism was related to cortical bone loss [31]. Our findings demonstrated that the increased femoral bone loss was accompanied by high serum FGF23, PTH, hyperphosphatemia, and hypercalcemia in nephrectomized BKO mice drinking PBS. These factors may be mediators responsible for skeletal disorder in thalassemic mice with CKD exposed to PBS.

## Conclusions

BKO mice had decreased cancellous and cortical bone volume but were resistant to nephrectomy-induced bone loss due to low circulating erythropoietin levels. Nephrectomy increased bone turnover in WT, resulting in cancellous and cortical bone loss. High phosphate loading had a negative correlation with cancellous and cortical bone in nephrectomized BKO mice, probably as a result of high circulating FGF23, hyperphosphatemia, and hyperparathyroidism.

## Supporting information

S1 Table Oligonucleotide primers for qPCR analysis. (DOCX)

S2 Table Hematological data of BKO and WT controls with nephrectomy and PBS. (DOCX)

S3 Table μCT analysis of BKO and WT controls with nephrectomy and PBS in femurs. (DOCX)

S4 Table Histomorphometric analysis of BKO and WT controls with nephrectomy and PBS in femurs. (DOCX)

S5 Table Summary results of two-way ANOVA. (DOCX)
