# Supplementary material for: High phosphate intake induces bone loss in nephrectomized thalassemic mice
Source: PLoS One. 2022 May 27;17(5):e0268732. doi: 10.1371/journal.pone.0268732 (PMC9140286; doi:10.1371/journal.pone.0268732)
Supplement: S1 Table — (DOCX) [file pone.0268732.s001.docx]

S1 Table. Oligonucleotide primers for qPCR analysis.

| Gene | Forward primer (5’ to 3’) | Reverse primer (5’ to 3’) |
| --- | --- | --- |
| *Alpl* | CTTGACTGTGGTTACTGCTGATCA | GTATCCACCGAATGTGAAAACGT |
| *Sp7* | CCCTTCTCAAGCACCAATGG | AAGGGTGGGTAGTCATTTGCATA |
| *Bglap* | GCTGCCCTAAAGCCAAACTCT | AGAGGACAGGGAGGATCAAGTTC |
| *Col1a1* | CCCAAGGAAAAGAAGCACGTC | ACATTAGGCGCAGGAAGGTCA |
| *Ibsp* | TGGCGACACTTACCGAGCTT | CCATGCCCCTTGTAGTAGCTGTA |
| *Kl* | AAGTCTTCGGCCTTGTTCTAC | CAAGCAAAGTCACAGGGAAATG |
| *Fgf23* | AGGACCAGCTATCACCTACA | CGAGTCATGGCTCCTGTTATC |
| *Sost* | ATCATTTCCAGACACCTCTTAC | ATGTGCTTCTGTTACAAACGCTC |
| *Ctsk* | AGGCATTGACTCTGAAGATGCT | TCCCCACAGGAATCTCTCTG |
| *Acp5* | GATCCCTCTGTGCGACATCA | CCAGGGAGTCCTCAGATCCA |
| *Ifng* | AAATCCTGCAGAGCCAGATTAT | GCTGTTGCTGAAGAAGGTAGTA |
| *Tnfa* | TTGTCTACTCCCAGGTTCTCT | GAGGTTGACTTTCTCCTGGTATG |
| *Sufu* | GTGCTATTGCCTTCCAAGCG | GCAGTGGGCTTTACCCTCTT |
| *Tnfsf11* | CAAGCTCCGAGCTGGTGAAG | CCTGAACTTTGAAAGCCCCA |
| *Tnfrsf11b* | AAGAGCAAACCTTCCAGCTGC | CACGCTGCTTTCACAGAGGTC |
| *Gapdh* | TGCACCACCAACTGCTTAG | GGATGCAGGGATGATGTTC |
