# Supplementary material for: High phosphate intake induces bone loss in nephrectomized thalassemic mice
Source: PLoS One. 2022 May 27;17(5):e0268732. doi: 10.1371/journal.pone.0268732 (PMC9140286; doi:10.1371/journal.pone.0268732)
Supplement: S2 Table — (DOCX) [file pone.0268732.s002.docx]

S2 Table. Hematological data of BKO and WT controls with nephrectomy and PBS.

| Parameters | WT | | | | BKO | | | | |
| --- | --- | --- | --- | --- | --- | --- | --- | --- | --- |
|  | Sham | Nephrectomy | Nephrectomy +PBS | Sham | | Nephrectomy | Nephrectomy +PBS | |  |
|  | (n=7) | (n=7) | (n=9) | (n=7) | | (n=4) | (n=5) | |  |
| RBC (x10^6^/ul) | 8.84±0.12 | 6.69±0.23^a^ | 6.65±0.17^a^ | 6.84±0.26^a^ | | 5.37±0.40^abcd^ | | 5.50±0.24^abcd^ |  |
| Hb (g/dl) | 14.00±0.33 | 10.16±0.29^a^ | 10.26±0.35^a^ | 7.54±0.18^abc^ | | 6.45±0.92^abc^ | | 5.98±0.28^abcd^ |  |
| Hct (%) | 43.56±0.36 | 30.67±0.84^a^ | 30.01±0.96^a^ | 26.33±1.44^ab^ | | 24.00±4.16^abc^ | | 18.38±1.47^abcde^ |  |
| MCV (fl) | 49.27±0.27 | 45.93±0.35 | 45.11±0.44 | 38.93±2.92^abc^ | | 43.78±4.66 | | 35.16±1.56^abce^ |  |
| MCH (pg) | 15.81±0.19 | 15.17±0.17 | 15.43±0.19 | 11.06±0.30^abc^ | | 11.88±0.98^abc^ | | 10.86±0.26^abc^ |  |
| MCHC (g/dl) | 32.13±0.56 | 33.04±0.27 | 34.20±0.43 | 29.14±1.73^abc^ | | 27.58±1.99^abc^ | | 31.04±0.92^c^ |  |
| RDW-CV (%) | 13.66±0.61 | 18.86±0.59 | 16.19±0.56 | 39.69±1.96^abc^ | | 32.78±5.87^abcd^ | | 36.74±2.75^abc^ |  |
| RDW-SD (fl) | 28.94±1.46 | 39.30±1.62 | 32.81±1.45 | 68.21±8.86^abc^ | | 62.78±15.00^abc^ | | 56.78±6.39^ac^ |  |

^a^*p*<0.05 versus sham WT, LSD *post hoc test*.

^b^*p*<0.05 versus nephrectomized WT

^c^*p*<0.05 versus nephrectomized WT with PBS

^d^*p*<0.05 versus sham BKO

^e^*p*<0.05 versus nephrectomized BKO
