# Supplementary material for: High phosphate intake induces bone loss in nephrectomized thalassemic mice
Source: PLoS One. 2022 May 27;17(5):e0268732. doi: 10.1371/journal.pone.0268732 (PMC9140286; doi:10.1371/journal.pone.0268732)
Supplement: S3 Table — (DOCX) [file pone.0268732.s003.docx]

S3 Table. μCT analysis of BKO and WT controls with nephrectomy and PBS in femurs.

| Parameters | WT | | | | BKO | | | |
| --- | --- | --- | --- | --- | --- | --- | --- | --- |
|  | Sham | Nephrectomy | Nephrectomy +PBS | Sham | | Nephrectomy | Nephrectomy +PBS |  |
|  | (n=8) | (n=12) | (n=9) | (n=7) | | (n=8) | (n=7) |  |
| Cancellous Bone |  |  |  |  | |  |  |  |
| BV/TV (%) | 12.79±1.45 | 9.75±0.75^a^ | 9.85±0.69^a^ | 8.92±0.67^a^ | | 9.98±0.67^a^ | 7.26±0.58^abce^ |  |
| Tb.Th (mm) | 0.046±0.002 | 0.044±0.001 | 0.039±0.001^ab^ | 0.042±0.001^a^ | | 0.044±0.002^c^ | 0.032±0.001^abcde^ |  |
| Tb.N (/mm) | 4.14±0.20 | 3.39±0.15^a^ | 3.57±0.14^a^ | 3.76±0.15 | | 3.57±0.18^a^ | 3.45±0.11^a^ |  |
| Tb.Sp (mm) | 0.235±0.016 | 0.293±0.014^a^ | 0.274±0.013 | 0.261±0.012 | | 0.280±0.017^a^ | 0.286±0.011^a^ |  |
| Conn.D (/mm^3^) | 142±15 | 120±9 | 148±11 | 111±10^c^ | | 122±13 | 141±3 |  |
| SMI (-) | 1.95±0.19 | 1.93±0.08 | 1.76±0.05 | 2.22±0.06^bc^ | | 1.94±0.10 | 1.89±0.07^d^ |  |
| BMD (mgHA/cm^3^) | 111±15 | 67±8 ^a^ | 64±7^a^ | 60±8 ^a^ | | 71±7^a^ | 32±8^abce^ |  |
| Cortical Bone |  |  |  |  | |  |  |  |
| BV/TV (%) | 41.86±0.75 | 39.98±1.14 | 41.03±0.71 | 37.49±0.59^ac^ | | 37.92±1.71^a^ | 31.82±1.16^abcde^ |  |
| Cross-sectional volume (mm^3^) | 1.18±0.05 | 1.11±0.03 | 1.10±0.03 | 1.19±0.01 | | 1.15±0.04 | 1.14±0.01 |  |
| Cortical volume (mm^3^) | 0.493±0.027 | 0.442±0.013^a^ | 0.449±0.012 | 0.447±0.007 | | 0.432±0.014^a^ | 0.364±0.012^abcde^ |  |
| Marrow volume (mm^3^) | 0.683±0.031 | 0.668±0.027 | 0.647±0.023 | 0.746±0.011^bc^ | | 0.716±0.041 | 0.781±0.019^abc^ |  |
| Cortical thickness (mm) | 0.176±0.005 | 0.163±0.005^a^ | 0.165±0.003 | 0.161±0.003^a^ | | 0.157±0.005^a^ | 0.133±0.005^abcde^ |  |
| BMD (mgHA/cm^3^) | 528±9 | 490±15 | 508±9 | 467±8^a^ | | 469±24 ^a^ | 385±16^abcde^ |  |

^a^*p*<0.05 versus sham WT, LSD *post hoc test*.

^b^*p*<0.05 versus nephrectomized WT

^c^*p*<0.05 versus nephrectomized WT with PBS

^d^*p*<0.05 versus sham BKO

^e^*p*<0.05 versus nephrectomized BKO
