# Supplementary material for: High phosphate intake induces bone loss in nephrectomized thalassemic mice
Source: PLoS One. 2022 May 27;17(5):e0268732. doi: 10.1371/journal.pone.0268732 (PMC9140286; doi:10.1371/journal.pone.0268732)
Supplement: S4 Table — (DOCX) [file pone.0268732.s004.docx]

S4 Table. Histomorphometric analysis of BKO and WT controls with nephrectomy and PBS in femurs.

| Parameters | WT | | | BKO | | | |
| --- | --- | --- | --- | --- | --- | --- | --- |
|  | Sham | Nephrectomy | Nephrectomy +PBS | Sham | Nephrectomy | Nephrectomy +PBS |  |
|  | (n=9) | (n=6) | (n=6) | (n=6) | (n=6) | (n=7) |  |
| Static Bone Parameters |  |  |  |  |  |  |  |
| BV/TV (%) | 13.66±0.85 | 9.68±1.13^a^ | 9.36±0.65^a^ | 8.52±0.46^a^ | 8.78±0.51^a^ | 6.05±0.59^abcde^ |  |
| Tb.Th (μm) | 41.30±1.72 | 37.45±2.18 | 35.70±0.66^a^ | 36.49±0.59^a^ | 36.89±1.20^a^ | 29.41±0.91^abcde^ |  |
| Tb.Sp (μm) | 266±14 | 367±36^a^ | 354±25^a^ | 399±28^a^ | 388±18^a^ | 479±41^abce^ |  |
| Tb.N (mm) | 3.30±0.13 | 2.57±0.21^a^ | 2.62±0.16^a^ | 2.34±0.12^a^ | 2.38±0.10^a^ | 2.05±0.17^abc^ |  |
| Ob.S/BS (%) | 8.12±0.26 | 12.64±1.06^a^ | 12.92±1.02^a^ | 6.33±0.32^abc^ | 7.49±0.59^bc^ | 4.41±0.43^abcde^ |  |
| N.Ob/B.Pm (/mm) | 7.69±0.34 | 12.47±0.85^a^ | 13.01±0.95^a^ | 6.92±0.67^bc^ | 8.21±0.81^bc^ | 5.67±0.56^abce^ |  |
| N.Ob/T.Ar (/mm^2^) | 50.80±3.10 | 62.55±3.02^a^ | 66.91±2.89^a^ | 32.17±3.41^abc^ | 38.62±3.22^abc^ | 22.54±1.78^abcde^ |  |
| Oc.S/BS (%) | 0.284±0.049 | 1.055±0.144^a^ | 0.856±0.124^a^ | 0.368±0.073^bc^ | 0.467±0.07^bc^ | 0.624±0.091^ab^ |  |
| N.Oc/B.Pm (/mm) | 0.190±0.032 | 0.652±0.081^a^ | 0.580±0.071^a^ | 0.296±0.052^bc^ | 0.332±0.044^bc^ | 0.428±0.067^ab^ |  |
| N.Oc/T.Ar (/mm^2^) | 1.24±0.19 | 3.23±0.33^a^ | 2.94±0.26^a^ | 1.41±0.29^bc^ | 1.55±0.17^bc^ | 1.71±0.23^bc^ |  |
| ES/BS (%) | 0.202±0.058 | 1.097±0.169^a^ | 0.957±0.148^a^ | 0.287±0.10^bc^ | 0.398±0.079^bc^ | 0.578±0.085^abc^ |  |
| Dynamic Bone Parameters |  |  |  |  |  |  |  |
| MS/BS (%) | 19.30±1.47 | 23.70±3.59 | 28.63±3.00^a^ | 15.12±1.50^bc^ | 14.05±2.12^bc^ | 8.64±1.62^abc^ |  |
| MAR (μm/day) | 0.89±0.04 | 1.13±0.06 | 1.00±0.09 | 0.65±0.16^bc^ | 0.79±0.07^b^ | 0.75±0.11^b^ |  |
| BFR/BS (µm^3^/µm^2^/year) | 62.77±5.80 | 98.37±15.39^a^ | 106.39±16.59^a^ | 32.32±6.36^abc^ | 42.35±8.34^bc^ | 22.87±4.07^abc^ |  |
| BFR/BV (%/year) | 299±34 | 429±52 | 518±94^a^ | 169±27^bc^ | 206±46^bc^ | 126±21^abc^ |  |
| BFR/TV (%/year) | 33.40±3.37 | 47.52±8.94^a^ | 54.99±7.13^a^ | 13.56±3.42^abc^ | 20.69±4.72^bc^ | 9.43±1.69^abc^ |  |

^a^*p*<0.05 versus sham WT, LSD *post hoc test*.

^b^*p*<0.05 versus nephrectomized WT

^c^*p*<0.05 versus nephrectomized WT with PBS

^d^*p*<0.05 versus sham BKO

^e^*p*<0.05 versus nephrectomized BKO
