# Supplementary material for: High phosphate intake induces bone loss in nephrectomized thalassemic mice
Source: PLoS One. 2022 May 27;17(5):e0268732. doi: 10.1371/journal.pone.0268732 (PMC9140286; doi:10.1371/journal.pone.0268732)
Supplement: S5 Table — (DOCX) [file pone.0268732.s005.docx]

S5 Table. Summary results of two-way ANOVA.

| Parameters | Two-way ANOVA | | | | | |
| --- | --- | --- | --- | --- | --- | --- |
|  | BKO | Nephrectomy | PBS | Interaction | |  |
|  |  |  |  | BKO* Nephrectomy | BKO*PBS |  |
| IFTA score | NS | *p* < 0.05 | *p* < 0.05 | NS | NS |  |
| Hematological Data |  |  |  |  |  |  |
| RBC (x10^6^/μl) | *p* < 0.05 | *p* < 0.05 | NS | NS | NS |  |
| Hb (g/dl) | *p* < 0.05 | *p* < 0.05 | NS | *p* < 0.05 | NS |  |
| Hct (%) | *p* < 0.05 | *p* < 0.05 | *p* < 0.05 | *p* < 0.05 | NS |  |
| MCV (fl) | *p* < 0.05 | NS | *p* < 0.05 | *p* < 0.05 | *p* < 0.05 |  |
| MCH (pg) | *p* < 0.05 | NS | NS | *p* < 0.05 | NS |  |
| MCHC (g/dl) | *p* < 0.05 | NS | *p* < 0.05 | NS | NS |  |
| RDW-CV (%) | *p* < 0.05 | NS | NS | *p* < 0.05 | NS |  |
| **μCT analysis** |  |  |  |  |  |  |
| Cancellous Bone |  |  |  |  |  |  |
| BV/TV (%) | *p* < 0.05 | NS | NS | *p* < 0.05 | NS |  |
| Tb.Th (mm) | *p* < 0.05 | NS | *p* < 0.05 | NS | *p* < 0.05 |  |
| Tb.N (/mm) | NS | *p* < 0.05 | NS | NS | NS |  |
| Tb.Sp (mm) | NS | *p* < 0.05 | NS | NS | NS |  |
| Conn.D (/mm^3^) | NS | NS | *p* < 0.05 | NS | NS |  |
| SMI (-) | NS | NS | NS | NS | NS |  |
| BMD (mgHA/cm^3^) | *p* < 0.05 | NS | *p* < 0.05 | *p* < 0.05 | NS |  |
| Cortical Bone |  |  |  |  |  |  |
| BV/TV (%) | *p* < 0.05 | NS | *p* < 0.05 | NS | *p* < 0.05 |  |
| Cross-sectional volume (mm^3^) | NS | NS | NS | NS | NS |  |
| Cortical volume (mm^3^) | *p* < 0.05 | *p* < 0.05 | NS | NS | *p* < 0.05 |  |
| Marrow volume (mm^3^) | *p* < 0.05 | NS | NS | NS | NS |  |
| Cortical thickness (mm) | *p* < 0.05 | NS | *p* < 0.05 | NS | *p* < 0.05 |  |
| BMD (mgHA/cm^3^) | *p* < 0.05 | NS | *p* < 0.05 | NS | *p* < 0.05 |  |
| **Histomorphometry** |  |  |  |  |  |  |
| Static Bone Parameters |  |  |  |  |  |  |
| BV/TV (%) | *p* < 0.05 | *p* < 0.05 | NS | *p* < 0.05 | NS |  |
| Tb/Th (μm) | *p* < 0.05 | NS | *p* < 0.05 | NS | NS |  |
| Tb.Sp (μm) | *p* < 0.05 | NS | NS | NS | NS |  |
| Tb.N (mm) | *p* < 0.05 | *p* < 0.05 | NS | *p* < 0.05 | NS |  |
| Ob.S/BS (%) | *p* < 0.05 | *p* < 0.05 | *p* < 0.05 | *p* < 0.05 | *p* < 0.05 |  |
| N.Ob/B.Pm (/mm) | *p* < 0.05 | *p* < 0.05 | NS | *p* < 0.05 | NS |  |
| N.Ob/T.Ar (/mm^2^) | *p* < 0.05 | *p* < 0.05 | NS | *p* < 0.05 | *p* < 0.05 |  |
| Oc.S/BS (%) | *p* < 0.05 | *p* < 0.05 | NS | *p* < 0.05 | NS |  |
| N.Oc/B.Pm (/mm) | NS | *p* < 0.05 | NS | *p* < 0.05 | NS |  |
| N.Oc/T.Ar (/mm^2^) | *p* < 0.05 | *p* < 0.05 | NS | *p* < 0.05 | NS |  |
| ES/BS (%) | *p* < 0.05 | *p* < 0.05 | NS | *p* < 0.05 | NS |  |
| Dynamic Bone Parameters |  |  |  |  |  |  |
| MS/BS (%) | *p* < 0.05 | NS | NS | NS | *p* < 0.05 |  |
| MAR (μm/day) | *p* < 0.05 | *p* < 0.05 | NS | NS | NS |  |
| BFR/BS (µm^3^/µm^2^/year) | *p* < 0.05 | *p* < 0.05 | NS | NS | NS |  |
| BFR/BV (%/year) | *p* < 0.05 | NS | NS | NS | NS |  |
| BFR/TV (%/year) | *p* < 0.05 | *p* < 0.05 | NS | NS | NS |  |
| Serum Biochemistry |  |  |  |  |  |  |
| Urea nitrogen (mg/dl) | NS | *p* < 0.05 | NS | NS | *p* < 0.05 |  |
| Creatinine (mg/dl) | NS | NS | NS | NS | NS |  |
| Phosphorus (mg/dl) | *p* < 0.05 | *p* < 0.05 | *p* < 0.05 | NS | *p* < 0.05 |  |
| Calcium (mg/dl) | *p* < 0.05 | NS | *p* < 0.05 | *p* < 0.05 | NS |  |
| Erythropoietin (pg/ml) | *p* < 0.05 | *p* < 0.05 | *p* < 0.05 | *p* < 0.05 | *p* < 0.05 |  |
| FGF23 (pg/ml) | *p* < 0.05 | NS | *p* < 0.05 | *p* < 0.05 | *p* < 0.05 |  |
| PTH (pg/ml) | NS | *p* < 0.05 | *p* < 0.05 | NS | NS |  |
